# Evaluation of *Mollugo oppositifolia* Linn. as cholinesterase and β-secretase enzymes inhibitor

**DOI:** 10.3389/fphar.2022.990926

**Published:** 2023-01-04

**Authors:** Bhaskar Das, Pardeep K. Bhardwaj, Nanaocha Sharma, Arnab Sarkar, Pallab Kanti Haldar, Pulok K. Mukherjee

**Affiliations:** ^1^ School of Natural Product Studies, Department of Pharmaceutical Technology, Jadavpur University, Kolkata, India; ^2^ Institute of Bioresources and Sustainable Development, Department of Biotechnology, Government of India, Imphal, India; ^3^ Department of Pharmaceutical Technology, Jadavpur University, Kolkata, India

**Keywords:** molecular docking, *Mollugo oppositifolia* Linn., Alzheimer’s disease, herb-drug interaction, safety evaluation, metabolite profiling and identification

## Abstract

*Mollugo oppositifolia* Linn. is traditionally used in neurological complications. The study aimed to investigate *in-vitro* neuroprotective effect of the plant extracts through testing against acetylcholinesterase (AChE), butyrylcholinesterase (BChE), and β-secretase linked to Alzheimer’s disease (AD). To understand the safety aspects, the extracts were tested for CYP450 isozymes and human hepatocellular carcinoma cell (HepG2) inhibitory potential. The heavy metal contents were estimated using atomic absorption spectroscopy (AAS). Further, the antioxidant capacities as well as total phenolic content and total flavonoid content (TFC) were measured spectrophotometrically. UPLC-QTOF-MS/MS analysis was employed to identify phytometabolites present in the extract. The interactions of the ligands with the target proteins (AChE, BChE, and BACE-1) were studied using AutoDockTools 1.5.6. The results showed that *M. oppositifolia* extract has more selectivity towards BChE (IC_50_ = 278.23 ± 1.89 μg/ml) as compared to AChE (IC_50_ = 322.87 ± 2.05 μg/ml). The IC_50_ value against β-secretase was 173.93 μg/ml. The extract showed a CC_50_ value of 965.45 ± 3.07 μg/ml against HepG2 cells and the AAS analysis showed traces of lead 0.02 ± 0.001 which was found to be within the WHO prescribed limits. Moreover, the IC_50_ values against CYP3A4 (477.03 ± 2.01 μg/ml) and CYP2D6 (249.65 ± 2.46 μg/ml) isozymes justify the safety aspects of the extract. The *in silico* molecular docking analysis of the target enzymes showed that the compound menthoside was found to be the most stable and showed a good docking score among all the identified metabolites. Keeping in mind the multi-targeted drug approach, the present findings suggested that *M. oppositifolia* extract have anti-Alzheimer’s potential.

## Introduction

Alzheimer’s disease (AD) is a neurodegenerative disorder and contributes major vote to dementia-related brain complications. The cholinergic hypothesis is mostly considered an important factor and is neurochemically characterized by consistent interruptions in cholinergic neurotransmission which specifically affect the basal forebrain (Chen and Mobley, 2019; Chen et al., 2022). Cumulative evidence suggests that cholinesterase inhibitors (AChE and BChE) can improve cholinergic transmission by blocking the degradation of choline, therefore, serving as a strategy for alleviating the symptoms of AD in patients (Moreta et al., 2021). Currently, donepezil or rivastigmine (AChE inhibitors) were used to alleviate cognitive function in patients with moderate to severe AD (Marucci et al., 2021). Besides, neuropathological changes in AD brains are also manifested by Aβ (beta-amyloid) senile plaques and neurofibrillary tangles deposition where β-site amyloid precursor protein cleaving enzyme (BACE) is the rate-limiting enzyme in the Aβ peptide production (Singh et al., 2016). In the pathway of amyloidogenesis amyloid precursor protein (APP), an integral membrane protein is cleaved by β-secretase and subsequently snipped by γ-secretase. Extracellular assembly of these cleaved protein fragments with intermediate composed into oligomers and protofibrils which has a stellar role in the development of insoluble senile plaques intern disrupting the neuronal networks. Inhibition of Aβ neurotoxic function also has a modifying effect on AD (Hampel et al., 2021). Despite substantial quantities of both forms (BACE-1 and BACE-2) of the β-secretase enzyme, Ahmed et al., 2010 confirmed that the BACE-1 subtype is the major target that could cease the formation of Aβ at the very beginning of APP processing. Verubecestat and LY3314814 are the only available drug candidates that progressed to Phase 3 clinical trials as β-secretase enzyme inhibitors (Zhao et al., 2020).

The multidisciplinary based approach plays a substantial role in the discovery of new drug candidates, for example, galanthamine is one most prevalent cholinesterase inhibitors that are being used clinically in the symptomatic relief of Alzheimer’s disease (Marucci et al., 2021). Physostigmine from *Physostigma venenosum* Balf. is another AChE inhibitor that provided a scaffold for the development of more effective cholinesterase inhibitors like rivastigmine (Berkov et al., 2021). Besides, there are several other drug candidates under different phases of the clinical trial for example Huperzine A from *Huperzia serrata* (Thunb.) Trevis. resveratrol from *Vitis vinifera* L. nicotine from *Nicotiana tabacum* L. curcumin from *Curcuma longa* L. (Ayaz et al., 2019).

BACE and cholinesterase inhibitors both of which have serious side effects and this might help to slow down the pathophysiological alterations and be unable to reverse the disease progression. To overcome this limitation, herbal medicine could be a better approach, since they constitute a different class of secondary metabolites targeting multiple signaling pathways or proteins, and show synergistic effects with minimum side effects (Sharma et al., 2017; Bhadra et al., 2022).

*Mollugo oppositifolia* Linn. (Molluginaceae family) is a glabrous, dioecious annual or perennial shrub traditionally used to treat cough, fever, inflammation, pain, jaundice, abdominal pain, intestine and urinary infections, appetite condition, malaria, viral infections, helminthiasis (AsokKumar et al., 2009; Sahu et al., 2012). This edible herb is locally known as Bakkhate in Mizoram, North-East India, and owns several therapeutic reputations (Khomdram et al., 2019). In Ayurveda, it is used to cure vata, piles, kapha, and leucoderma (Hoque et al., 2011a; 2011b; Sahu et al., 2012). Different solvent extracts of the plant parts were reported to have antioxidant, antimicrobial, anti-diabetic, sedative, and anxiolytic activities (Martin-Puzon et al., 2015; Tanquilut et al., 2019; Zhang et al., 2019; Nagannawar and Jayaraj, 2020).

The plant was reported to contain oleanolic acid; lupeol; dodecanoic acid; heptanoic acid; hexadecanoic acid; stigmasterol; isobornyl acetate; 8,11,14-eicosatrienoic acid (Nagannawar and Jayaraj., 2018 and, 2020 Modi and Shah, 2015). Besides this the plant also contains benzoic acid; 4-hydroxybenzoic acid; vanillin; cinnamic acid; vitexin; spergulacin; spergulagenin-A; linoside A; spergulin A & B; spinasterol, β-sitosterol, stigmasterol; squalene (Traore et al., 2000; Ragasa et al., 2012; Ha et al., 2021).

The present study explores the inhibitory activity of the *M. oppositifolia* extract against cholinesterase, β-secretase and cytochromeP450 enzymes together with the assessment of antioxidant capacity, TPC, TFC, heavy metal contents, and cytotoxicity potentials. The UPLC-QTOF-MS analysis combined with molecular docking study was performed to access the interaction potential of the phytocompounds towards target enzymes.

## Materials and methods

### Procurement of plant material and extraction

Aerial parts of *M. oppositifolia* were collected from a local vegetable market, Jadavpur, Kolkata (West Bengal), and the voucher specimen (IBSD/22/M-001) was maintained at the Institute of Bioresources and Sustainable Development, Manipur, Imphal, India for future reference. The plant sample was cleaned and shade dried till constant weight was attained. Accurately weighed 100 g grounded coarsely powdered crude plant sample was macerated using 500 ml of 80% methanol for 72 h. Then the mixture was filtered through Whatman filter paper (nylon; 0.45 µm) and the filtrate was concentrated by a rotary vacuum evaporator (IKA, Japan). The lyophilized (Instrumentation India, India) extract was packed in a sealed container and stored in the freezer (4°C). The percentage yield was 10.54% w/w.

### Chemicals and reagents

AChE (C3389), BChE (C1057), ATCI (01480), BTCI (20820), DTNB (D218200), galanthamine (G1660), ketoconazole (UC280), and fluoxetine (F132) were purchased from Sigma-Aldrich (Steinheim, Germany). β-secretase Activity Assay Kit (ab65357) purchased from Abcam (Cambridge, United Kingdom). Vivid^®^ CYP450 Screening Kit [CYP2D6 (P2972) and CYP3A4 (P2858)] were purchased from Life Technologies (United States). Methanol (≥99.8%) LC grade (Merck-Mumbai, India), 0.45 μ syringe filter (Millipore, Germany). Stock solutions for all the metals (1,000 ppm) were procured from Merck (Darmstadt, Germany). HepG2 cells were obtained from the American Type Culture Collection (ATCC HB 8065). Dulbecco’s modified Eagle’s medium (DMEM), phosphate Buffer Saline (PBS), and fetal bovine serum (FBS) were purchased from Life Technologies Ltd. (Fairlands, Johannesburg, RSA). Dimethyl sulfoxide (DMSO), trypsin, deoxyribose, thiobarbituric acid (TBA), trichloroacetic acid (TCA), MTT, EDTA, FeCl_3_, H_2_O_2_, NaOH, HNO_3_, HClO_4_, HCl, and H_2_SO_4_ and other reagents were analytical grade (Merck, Germany), All the working solutions were freshly prepared on the day of analysis.

The cell line was maintained in DMEM supplemented with 10% FBS, non-essential amino acids, and sodium pyruvate (1 mM). The cell line was grown at 37°C in a humidified incubator set at 5% CO2 and sub-cultured with 0.25% (w/v) trypsin and 0.53 mM EDTA for a maximum of 15 min every 2–3 days after they had 90% confluent monolayer.

## Evaluation of free radical scavenging potential

### DPPH radical scavenging assay

The assay was performed in a 96-well microplate as described by Das et al., 2020. The test wells contain 100 μL of the extract solution (50–300 μg/ml) and 100 μL of 0.1 mM DPPH radical solution in methanol. After 30 min of incubation in the dark, the absorbances were measured at 517 nm using a Spectramax iD3 reader (CA, United States). at 28°C. In the positive control group ascorbic acid (AA) was used as the reference standard.

The % inhibitions were calculated using the formula:

$$\%\;inhibition=\left[\frac{\left(A1-A0\right)}{A1}\right]\times 100$$

(Where A1 and A0 are the absorbances of the control and test samples respectively).

### Hydroxyl radical scavenging assay

The assay was performed with slight modifications to the method described by Das et al., 2020 in a 96-well microplate A reaction mixture containing 10 μL of deoxyribose (28 mM) in phosphate buffer (50 mM, pH 7.4), 10 μL of FeCl_3_ (1 mM), 10 μL of EDTA (1 mM), 10 μL of H_2_O_2_ (1 mM) was prepared. To the test and positive control wells 100 μL each of the extract and ascorbic acid (reference standard) dissolved in methanol (50–300 μg/ml) were added. After 1 h of incubation at 42°C, the reaction was stopped by adding 50 μL of 50 μL of 0.5% TBA and 10% TCA in NaOH solution (50 mM) and incubated over again for 30 min at 42°C. Similarly, a control was prepared. The absorbances were recorded after 30 min of incubation at 42°C at 532 nm using a Spectramax iD3 reader (CA, United States).

The radical scavenging capacity was calculated using the formula:

$$\%\;inhibition=\left[\frac{\left(A1-A0\right)}{A1}\right]\times 100$$

(Where A1 and A0 are the absorbances of the control and test samples respectively).

### Nitric oxide radical scavenging assay

The assay was performed as described by Das et al., 2020 using a 96-well microplate. To 1 ml of the extract and ascorbic acid (30–500 μg/ml) dissolved in methanol 0.5 ml of sodium nitroprusside in phosphate buffer saline (10 mM) was mixed and incubated for 180 min at 25°C. In the test and positive control wells 150 μL of the reaction mixture containing the extract or standard drug were transferred followed by the addition of 150 μL of freshly prepared Griess reagent (1% sulphanilamide in 2.5% phosphoric acid, and 0.1% naphthyl ethylene diamine dihydrochloride in 2.5% phosphoric acid in equal ratio) was transferred to 96-well microplate. Similarly, a control group was also prepared. The absorbance of the solutions was recorded at 546 nm using SpectraMax iD3 reader (CA, United States).

The % inhibitions were calculated using the following formula:

$$\%\;inhibition=\left[\frac{\left(A1-A0\right)}{A1}\right]\times 100$$

(Where A1 and A0 are the absorbances of the control and test samples respectively).

### Determination of total phenolic and flavonoid contents

The total phenolic content (TPC) and total flavonoid content (TFC) present in the extract were determined spectrophotometrically as described by Pieczykolan et al., 2022. Stock solutions of the test extract and standards were prepared in methanol.

For TPC, 0.5 ml of the test extract or standard stock solutions were taken and to this 2.5 ml of 10%, Folin-Ciocalteu’s reagent and 2.5 ml of 7.5% NaHCO_3_ were added. In the same way, a blank was also prepared. Gallic acid (10–40 μg/ml) was used as the standard to prepare the calibration curve. The reaction mixtures were incubated for 45 min at 45°C and the absorbance was recorded at λ_max_ 765 nm using SpectraMax iD3 reader (CA, United States). The experiment was performed in triplicate. The content of total phenolics present in the extract was expressed as gallic acid equivalent (mg of GAE/g of extract).

For TFC, 1 ml of the test extract or standard stock solutions and 1 ml of 2% AlCl_3_ solution in methanol were mixed. Similarly, a control was also prepared. Rutin (20–80 μg/ml) was used to prepare the calibration curve For the construction of the calibration curve. After 1 h of incubation at 28°C, absorbance was measured at λ_max_ 415 nm. All the samples were analyzed in triplicate. The content of total flavonoids in the extract was expressed as rutin equivalent (mg of RUE/g of extract).

## Evaluation of anti-Alzheimer’s potential by an *in-vitro* method

### Cholinesterase enzyme inhibition study potential

The method for cholinesterase enzyme inhibitory activity of the extract was performed following the method described by Das et al., 2020. The wells are grouped as background, test, and positive control. Each well of the plate contained 125 μL of 3 mM DTNB and 25 μL of 15 mM ATCI or BTCI. The background and test wells contained 25 μL of the extract (50–300 μg/ml) dissolved in tris buffer (_P_H 8.0). To the positive control well, 25 μL of galanthamine (10–50 μg/ml) dissolved in tris buffer (_P_H 8.0) was transferred. After 10 min of pre-incubation at 37°C, 25 μL of 2 U/ml AChE or 1 U/mL BChE were added in each well except background wells, and the absorbance was recorded at 405 nm for every 13 s up to 78 s. The linear regression parameters were determined for each curve. The %inhibition of the enzymatic activity was calculated and the IC_50_ values were extrapolated. The assay was performed in triplicate (*n* = 3).

### β-secretase enzyme inhibitory potential

The *in-vitro* β-secretase enzyme inhibitory activity of the plant extract was performed following the method described by Mooko et al., 2021. The wells are grouped as blank, test, positive control, and negative control. The control as well test wells contained 50 µL of the extract (7.8125–500 μg/ml) dissolved in extraction buffer. The positive control well contained 50 µL of the extraction buffer and 2 µL of active β-secretase enzyme. To the negative control well 50 µL of the extraction buffer, 2 µL of the active β-secretase enzyme, and 2 µL of β-secretase inhibitor were added. Then 50 µL of the reaction buffer was added to each well. After 10 min of incubation at 37°C, 2 µL of β-secretase substrate was added to each well except the control wells. The plate was gently shaken and incubated again at 37°C for 60 min in dark conditions. The fluorescence activity was measured using a fluorescence microplate reader (PHERAstar, BMG Labtech, United States) using an excitation wavelength of 335 nm and an emission wavelength of 460 nm. The assay was performed in triplicate and IC_50_ values were calculated accordingly.

### *In vitro* cytotoxicity assessment

The toxic effect of the extracts in HepG2 cells was performed *via* MTT cytotoxicity assay as described by Das et al., 2022. HepG2 cells were cultured and maintained in DMEM supplemented with 10% (v/v) FBS, l-glutamine (4 mM), penicillin (100 U/mL) and streptomycin (100 μg/ml). In each well of the 96-well microtiter plate, 100 µL of the media containing cell suspension (1×10^4^ cells/mL) was seeded and incubated at 37°C with 5% CO_2_ for 24 h to allow the cells to attach to the bottom of the wells. Subsequently, cells were exposed to 100 μL each of the extract solution (100–1,000 μg/ml), controls which included vehicle-treated cells exposed to 0.5% DMSO and exposed to positive inhibitor doxorubicin (0.78125–100 μg/ml). After 24 h the cells were exposed to the МТТ reagent (0.5 mg/ml) and the absorbance was measured at 570 nm using a plate reader (Multiskan Go, Thermo Fischer Scientific). Color control blanks were utilized to normalize the results and the vehicle control treated cells were considered as 100% cell viability. The results are representative of the average % inhibition of all the experiments three times repeated and given as CC_50_.

### CytochromeP450 enzymes inhibition assay

A fluorogenic assay was performed in black 96-well microplates following the method described by Das et al., 2022. The test extract, 10 μM ketoconazole (CYP3A4), and 10 μM fluoxetine (CYP2D6) were dissolved in 100 mM Vivid CYP450 reaction buffer and diluted in 2-fold serial dilution. To each well 40 μL of the test extract or standard inhibitors and 50 μL of prepared master pre-mix (containing reaction buffer, P450 Baculosomes Plus Reagent, and Regeneration System) were added and pre-incubated for 20 min at 37°C. Inhibition potential was tested against CYP3A4 (5 nM), and CYP2D6 (10 nM) isozymes. The reaction was started by the addition of 10 μL of NADP+ and the appropriate substrate (10 μM concentrations of fluorogenic substrates namely, 7-benzyloxymethyloxy-3-cyanocoumarin and 7-ethoxymethoxy-3-cyanocoumarin were used for CYP3A4 and CYP2D6 respectively) mixture in reaction buffer and again incubated for 10 min at 37°C. The fluorescence activity was measured using a fluorescence microplate reader (PHERAstar, BMG Labtech, United States) with an excitation wavelength of 415 nm and an emission wavelength of 460 nm. Accordingly, percentage inhibition and IC_50_ value were determined.

### Estimation of heavy metals content

Heavy metals content estimation was performed using atomic absorption spectroscopy (Thermo Fisher AA 303; Thermo Fisher, Nasik, Maharashtra, India). Accurately weighed 1.0 g of the dried extract was treated with 3 ml of conc. HNO_3_ for 4–5 h. To this 3 ml of the acid mixture containing HNO_3_:HClO_4_ (2:1) was added and heated for 5–6 h at 120°C–130°C until the fumes stop and the solution became clear. At that point, 10 ml of milli-Q water was added and again boiled for 10–15 min till the volume was reduced to half. The resulting solution was cooled to room temperature and filtered using Whatman filter paper. The filtrate volume was adjusted to 50 ml with Milli-Q water. Similarly, a blank was also prepared (Nema et al., 2014). The experiment was repeated three times. For the analysis of the heavy metals, the instrumental condition is maintained as mentioned in Supplementary Table S1.

### Chemical profiling by UPLC-QTOF-MS

Tentative identifications of the compounds were performed using Waters UPLC hyphenated with a Waters Synapt G2 QTOF instrument. Analysis was performed using an Acquity UPLC BEH C18 1.7 daysµm (2.1 × 100 mm column) column operating at a 0.300 ml/min flow rate. For analysis accurately weighted 1 mg of the extract was dissolved in 50% methanol (LC grade) and filtered through a 0.22 µm syringe filter. The mobile phase used was: A, 0.1% HCO_2_H in LC grade water and B, MeOH + 0.1% HCO_2_H. MS source ESI operated in both positive and negative ionization modes, capillary voltage and endplate voltage were set at 2600V and 2000 V respectively. Nitrogen was used as nebulizing gas at 10 L/h and m/z range was set from 50 to 1,200 amu. Gradient elution started with 3% B which remain linear until 14 min. From 14 to 16 min elution was kept constant with 100% B. A linear gradient of 3% B was then used to reach completion until 20 min. The mass data were processed by integrating with MassLynx v 4.1 (Waters Corporation, Milford, MA, United States), TagetLynx (Waters Corporation, Milford, MA, United States) and Openchrom (Eclipse Foundation Inc. Ottawa, Canada) software, which provided a list of possible elemental formulae.

## Molecular modeling studies

### Protein preparation

The Protein Data Bank (https://www.rcsb.org) was used to obtain the 3D crystal structures of AChE (PDB ID: 4M0E), BChE (PDB ID: 5K5E), and BACE 1 (PDB ID: 6OD6) with resolutions of 2.0, 2.8, and 2.0 Å respectively. Discovery Studio Visualizer v21.1.020298 was used to eliminate the complexes, inhibitors, non-essential water molecules, and all heteroatoms. On AutoDockTools 1.5.6, the macromolecule preparation procedure for docking studies was followed. Gasteiger charges were added after non-polar hydrogens were merged. The eliminated complex structures were then applied to undergo docking assessments (Zilbeyaz et al., 2021).

### Ligand preparation

PubChem (https://pubchem.ncbi.nlm.nih.gov) was used to retrieve the three-dimensional structure of six small compounds discovered using LC-MS data in. sdf format. The mmff94 technique was used to achieve energy minimization for those molecules. This force field technique optimized the ligand conformer, resulting in a low-energy conformer. The ligand molecules were also added with hydrogen atoms, and their bond orders were fixed. PyRx was used to carry out a molecular docking study based on the conformation of the ligands with the lowest energy (Latha and Saddala, 2017; Lakshmanan et al., 2019).

### Molecular docking

The interactions of the ligands with the target proteins were studied using molecular docking simulations. The docking was done with the PyRx 0.8 docking tool, utilizing a grid-based approach (−17.1948771822; −42.3786708589; 25.5801503824 for AChE; −3.3503745278; 9.52083690694; 14.4095583015 for BChE and −36.9335726532; −45.3980852787; 19.5144221109 for BACE-1) and the rigid dock strategy with the AutoDock Vina inbuilt algorithm were used to calculate the binding energy (Kcal/mol) of all protein-ligand complexes. Discovery Studio Visualizer was used to investigate the characteristics of the protein-ligand complex and their interactions. The hydrogen bond interactions with amino acids, π-Alkyl, π-π, π-σ interactions, and distance were estimated after the binding site was seen (Abdel-Hamid and McCluskey, 2014; Dallakyan and Olson, 2015).

### Statistical analysis

The experimental data were expressed as mean ± SEM of triplicate. The significance of the difference between the groups was assessed by an unpaired *t*-test. The statistical significance was calculated using GraphPad Prism 5.0, a *p* < 0.05 was considered to be statistically significant.

## Results

### Evaluation of free radical scavenging potential

The antioxidant potential of *M. oppositifolia* was performed spectrophotometrically. The respective IC_50_ value was determined from the calibration curve after plotting varying concentrations against %inhibition. The IC_50_ values were found to be 93.21 ± 2.16 and 138.24 ± 1.41 μg/ml for ascorbic acid (AA) and *M. oppositifolia* extract, respectively by the DPPH method. The IC_50_ values for hydroxyl radical scavenging activity were 98.73 ± 1.96 and 189.43 ± 1.69 μg/ml for ascorbic acid (AA) and *M. oppositifolia* extract. For the nitric oxide radical scavenging assay, the IC_50_ values were 78.9 ± 0.63 and 264.32 ± 1.02 μg/ml for ascorbic acid (AA) and *M. oppositifolia* extract, respectively.

### Determination of TPC and TFC

The TPC and TFC present in *M. oppositifolia* extract were expressed in terms of gallic acid equivalent (*y* = 0.0084x + 0.0055; r^2^ = 0.92) and rutin equivalent (*y* = 0.0029x–0.0051; *r* ^2^ = 0.98). The concentration of total phenols and flavonoids were found to be 11.23 ± 0.56 mg of GAE/g of extract and 21.51 ± 0.64 mg of RUE/g of extract respectively.

### Assessment of anti-Alzheimer’s potential by *in-vitro* methods

**FIGURE 1:**
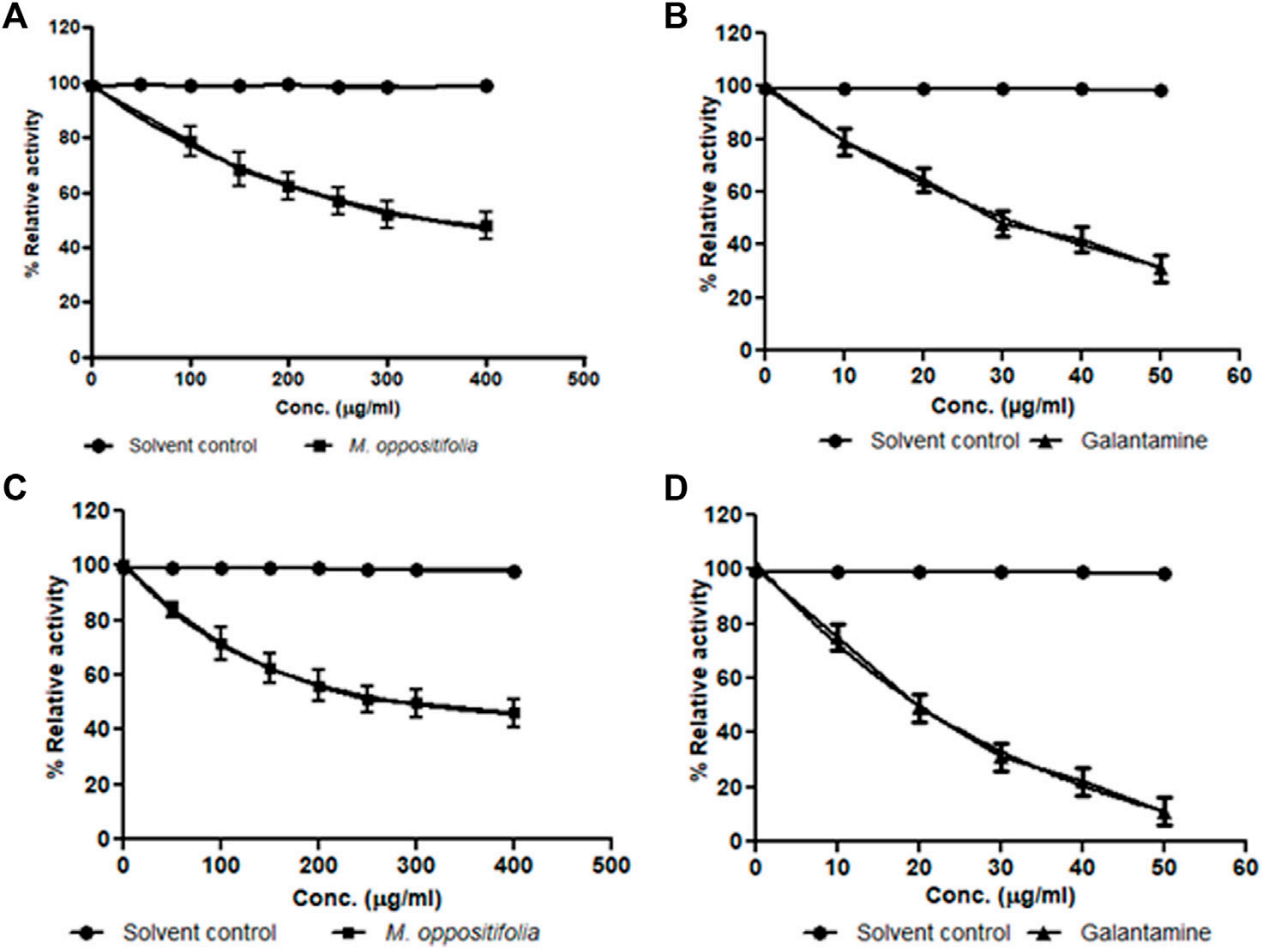
Dose-response curve for Solvent control (•); *M. oppositifolia* (■) and galanthamine (▲) for **(A,B)** acetylcholinesterase and **(C,D)** butyrylcholinesterase enzyme inhibition.

The cholinesterase enzyme inhibitors are a significant therapeutic target and are considered first-line pharmacotherapeutics for AD. The *in-vitro* cholinesterase enzyme inhibition activity of the test material and the standard were expressed as IC_50_ values. The IC_50_ values for the AChE inhibitory activity of *M. oppositifolia* extract and galanthamine (Standard inhibitor) were found to be 322.87 ± 2.05 and 32.71 ± 0.57 μg/ml. The IC_50_ values for BChE inhibition were 278.23 ± 1.89 and 20.15 ± 0.16 μg/ml respectively (Figure 1).

**FIGURE 2:**
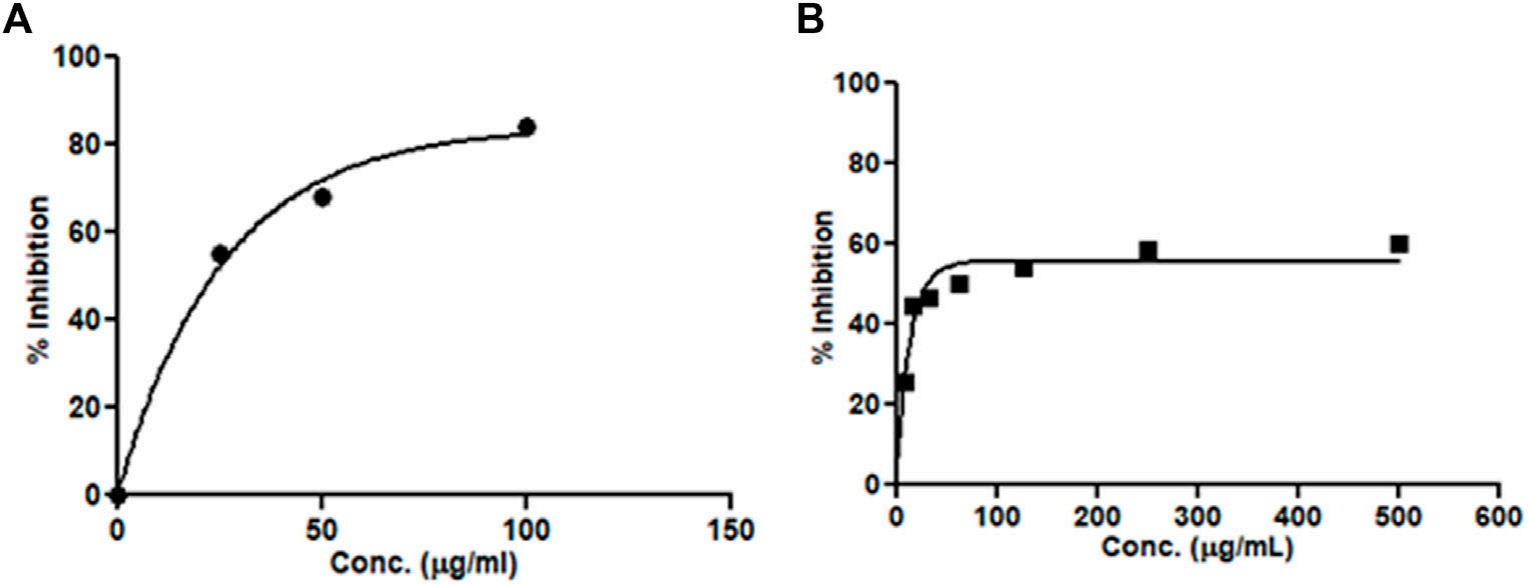
Concentration-dependent β-secretase enzyme inhibitory activity by **(A)** standard inhibitor and **(B)** *M. oppositifolia* extract.

The current treatment in AD is aimed at lowering β-amyloid plaque accumulation in the cerebellum. The effect of the extracts on the β-secretase enzyme was determined through a β-secretase assay kit using the fluorescence method. The hydro-alcoholic extract of *M. oppositifolia* inhibited the β-secretase enzyme with an IC_50_ value of 173.93 μg/ml and the standard inhibitor showed a value of 41.09 μg/ml (Figure 2). The results suggested the possibility of *M. oppositifolia* extract in reducing or preventing the formation of the β-amyloid peptides.

## Safety assessment

### Cytotoxicity assessment in HepG2 cell line

The results from the *in-vitro* cytotoxicity assay showed that *M. oppositifolia* extract has a CC_50_ value of 965.45 ± 3.07 μg/ml whereas the standard cytotoxic agent doxorubicin showed much more toxic against HepG2 cells with CC_50_ value of 5.25 ± 0.24 μg/ml at 24 h. The results indicate that the test material is the least toxic in the HepG2 cell line.

### CYP450 isoenzymes inhibition potential

To understand the interaction potential of the extract with drug metabolizing enzymes *M. oppositifolia* extract was tested with different cytochromeP450 isozymes. Respective positive controls (ketoconazole for CYP3A4 & fluoxetine for CYP2D6) for each isozyme were used to confirm the assay precision.

**FIGURE 3:**
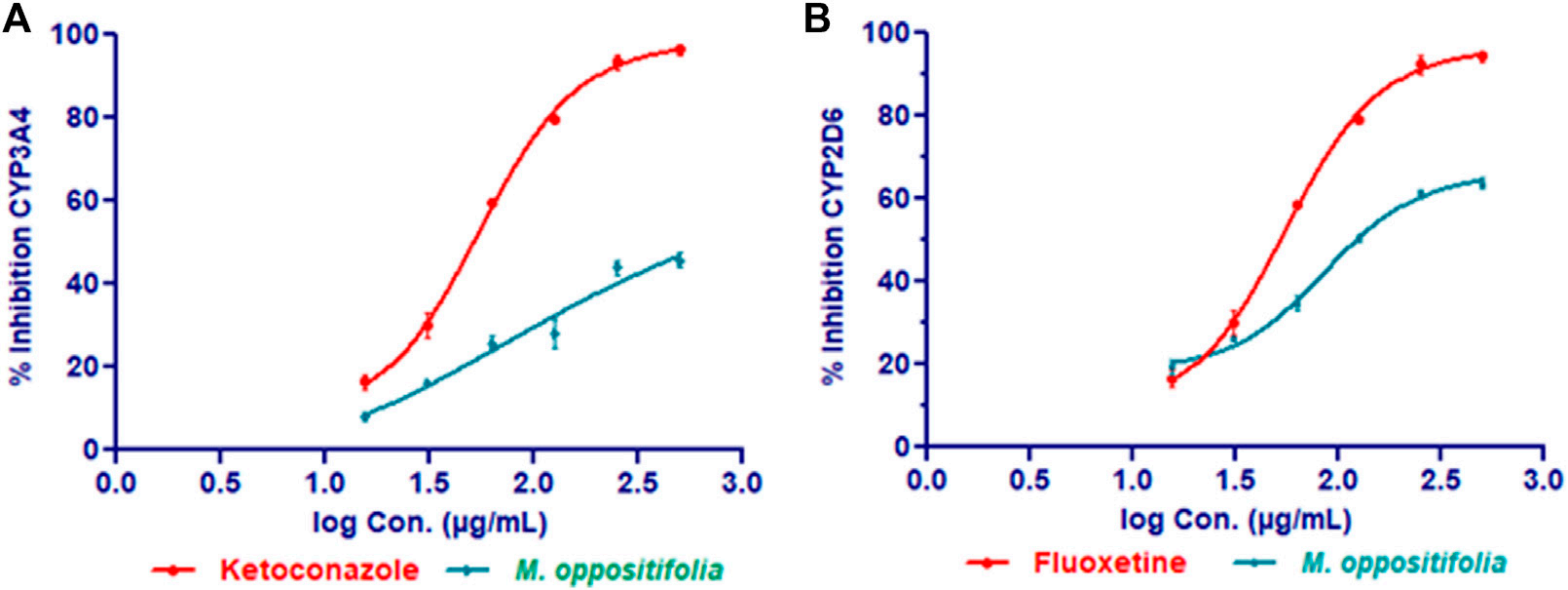
CYP3A4 **(A)** and CYP2D6 **(B)** inhibitory effect of *M. oppositifolia* and standard inhibitors.

The IC_50_ values of the *M. oppositifolia* extract and standards against CYP3A4 and CYP2D6 isozymes are represented in Figure 3. Against CYP3A4 and CYP2D6 the IC_50_ values were found to be 477.03 ± 2.01 and 249.65 ± 2.46 μg/ml of the extract and for the standard IC_50_ values were 6.66 ± 1.87 (ketoconazole) and 7.93 ± 1.56 μg/ml (fluoxetine) respectively.

### Quantitative estimation of heavy metals content by atomic absorption spectroscopy

In the present study, the concentration of heavy metals was determined using the standard calibration curve of the standard heavy metals solution having optimal detectable concentration ranges. The toxic heavy metals concentration was documented in terms of parts per million (ppm) as shown in Table 1.

**TABLE 1:** Heavy metals content in *M. oppositifolia* determined by AAS.

Plant	Heavy metal concentration (ppm)		
	Cd	Pb	Hg
*M. oppositifolia*	0.000	0.02 ± 0.001	0.000

### Chemical profiling by UPLC-QTOF-MS

**FIGURE 4:**
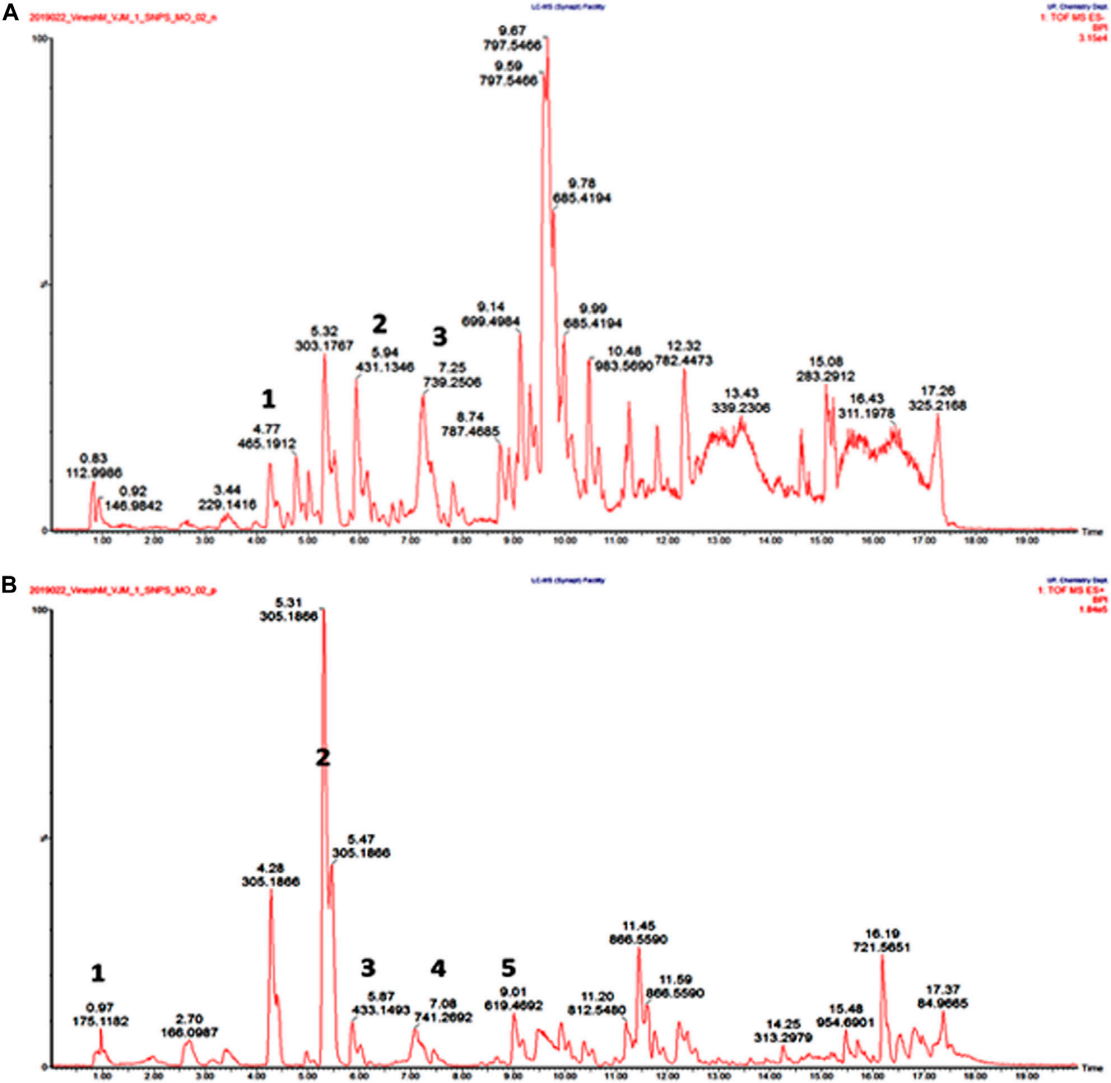
UPLC-QTOF-MS/MS total ion chromatogram for *M. oppositifolia* extract **(A)** and **(B)** represent positive and negative ionization modes, respectively.

Tentative identification of compounds present in the hydro-alcohol extracts of *M. oppositifolia* was carried out using Waters Synapt G2 QTOF in both negative and positive ionization modes generating the accurate mass or quasimolecular ion and formula. The total ion chromatograms and full MS reports of the extracts are provided in Figure 4; Table 2, respectively.

**TABLE 2:** UPLC-QTOF-MS profiles of *M. oppositifolia* hydro-alcohol extract (analysis in both negative and positive mode).

Source	Peak no	Rt (min)	Compound name	Chemical formula	Acquired (m/z)	Theoretical (m/z)	Literature references
Negative mode							
	1	4.77	Etiocholanolone glucuronide	C_25_H_38_O_8_	465.1912	466.2566	Anizan et al., 2011
	2	5.94	Genistin	C_21_H_20_O_10_	431.1348	432.1056	Chang et al. (2013)
	3	7.25	Menthoside	C_36_H_36_O_17_	739.2506	740.1952	Bensky, 2015
Positive mode							
	1	0.97	Caffeine	C_8_H_10_N_4_O_2_	175.1182	194.0809	Nemzer et al. (2021)
	2	5.31	Eugenyl glucoside	C_16_H_22_O_7_	305.1866	326.1365	Takeda et al. (1998)
	3	5.87	Unidentified		433.1493		
	4	7.08	Menthoside	C_36_H_36_O_17_	740.2692	740.1952	Bensky, 2015
	5	9.01	Ziyuglycoside II	C_35_H_56_O_8_	619.4692	604.3975	Jang et al. (2013)

A total of 07 compounds from 07 different chemical classes like steroids, isoflavones, flavonoids, methylxanthines, triterpenoid saponin, anthraquinone, and phenolic glycosides were characterized (Figure 4). A steroid glucosiduronic acid namely etiocholanolone glucuronide was detected at Rt 4.6 min showing molecular ion [M-H]- at m/z values at 465.1912 (Anizan et al., 2001). Genstin, a flavonoid derivative at 5.94 min produces molecular ion [M-H]- at m/z 431.1348 (Chang et al., 2013). Another flavonoid class of compound at Rt 7.25and 7.08 showed molecular ion [M-H]- and [M + H]+ at m/z 739.2506 and 740.2692 identified to be Menthoside (Bensky et al., 2015). Caffeine, a methylxanthine class of compound was identified at Rt. 0.97 showing [M + H]+ at m/z 175.1182 (Nemzer et al., 2021). A phenolic glycoside, Eugenyl glucoside was detected at Rt. 5.31 showing [M + H]+ at m/z 305.1866 (Takeda et al., 1998). A triterpenoid saponin namely Ziguglycoside II was detected at Rt. 9.01 showing [M + H]+ at m/z 619.4692 (Jang et al., 2013).

### Molecular docking study against AChE, BChE, and BACE-1 enzymes

The ligand–enzyme complexes with the compounds were placed in the same pocket of the AChE, BChE, and BACE-1 enzymes by PyRx 0.8.

The docking study of the compounds against AChE (PDB ID: 4M0E) possessed good docking scores ranging from −6.5 to −11.1 (Supplementary Figure S1; Supplementary Table S1). The most stable was menthoside with a docking score of -11.1 followed by genistin, while the least stable among the six compounds was caffeine. The reference standard compound galanthamine showed a docking score of −7.6. Other than Caffeine, all the compounds were found to interact *via* multiple hydrogen bonds with different amino acids in the active site of the macromolecule. Furthermore, other than conventional hydrogen bonds, the ligands also possessed different π interactions, including π-π stacked, π-σ, and π-alkyl interactions. The crucial amino acids within the active site that are mostly found to interact with the ligand molecules are TRP286, TYR124, and TYR341 residues (Supplementary Figure S2). The compound menthoside possessed conventional hydrogen bonds with VAL340, TRP286, SER293, and π -interactions with TYR72, HIS287, and TRP286 residues in the active site. The standard reference compound galanthamine was found to interact with SER293 *via* conventional hydrogen bonds by accepting the oxygen atoms, and also showed π -interactions with PHE297, PHE338, TRP286, TYR341 residues in the active site.

The docking study of the compounds against BChE (PDB ID: 5K5E) showed good docking scores ranging from -6.6 to -11.3 (Supplementary Figure S3; Supplementary Table S3). The most stable was menthoside with a docking score of −11.3 followed by ziyuglycoside II, while the least stable among the six compounds was caffeine. The reference standard compound galanthamine showed a docking score of −9.4. Other than Caffeine, all the compounds were found to interact *via* multiple hydrogen bonds with different amino acids in the active site of the macromolecule. Furthermore, other than conventional hydrogen bonds, the ligands also possessed different π interactions, including π-π stacked, π-σ, π-cation, and π-alkyl interactions. The crucial amino acids within the active site that are mostly found to interact with the ligand molecules are HIS438, GLY116, and TRP82 residues (Supplementary Figure S4). On the other hand, Menthoside possessed conventional hydrogen bonds with GLY115, GLY283, TYR282, ASN289, and π-interactions with HIS438, TRP82, and TYR332 residues. The compound galanthamine shared conventional hydrogen bonds with SER198, GLY115, GLY116, GLY117, and π -interactions with TRP82 residues in the active site.

The docking study of the compounds against BACE-1 (PDB ID: 6OD6) also possessed good docking scores ranging from −5.1 to −9.8 (Supplementary Figure S5; Supplementary Table S4). The most stable was menthoside showed a docking score of -9.8 followed by ziyuglycoside II, while the least stable among the six compounds was caffeine. All the compounds were found to interact *via* multiple hydrogen bonds with different amino acids in the active site of the macromolecule. Furthermore, other than conventional hydrogen bonds, the ligands also possessed different π interactions, including π-π stacked, π-σ, amide-π stacked, and π-alkyl interactions. The crucial amino acids within the active site that are mostly found to interact with the ligand molecules are PHE108, GLN73, ILE118, GLY34, TYR71, and TYR198 residues (Supplementary Figure S6). On the other hand, Menthoside possessed conventional hydrogen bonds with ARG128, SER36, PHE108, ASP228, and π-interactions with VAL69, ARG128, and TYR71 residues.

## Discussion

The brain is a much metabolically active body part and the polyunsaturated fatty acids contents are highly susceptible to oxidative damage. The detrimental role of ROS in AD initiation and progression results from higher Aβ production and accumulation of plaque (Cobley et al., 2018; Singh et al., 2019; Sharma and Kim, 2021). Thus antioxidants may be more effective to prevent and reverse AD progression (Gezici et al., 2020; Veurink et al., 2020). Phenols and flavonoids are considered potential natural antioxidants having the ability to scavenge free radicals and reactive oxygen species because of their conjugated ring structures and OH group’s substitution (Lopa et al., 2021). A study by Wang et al., 2014 suggested that treatment of the polyphenols combination resulted in improved protection against cognitive impairments in mice. Similarly, the presence of a substantial amount of phenolics and flavonoids in the hydro-alcoholic extract of *M. oppositifolia* suggests that it could considerably improve the care of patients with AD.

Currently, the US-FDA and Health Canada-approved acetylcholinesterase and NMDA antagonists alleviate cognitive functions in AD. Drugs like donepezil, rivastigmine, and galanthamine were the available drug candidate as cholinesterase inhibitors which might help to slow down the pathophysiological alterations of clinical symptoms, but not be able to reverse the disease progression (Grossberg et al., 2019).

The current treatment in AD is also aimed at lowering β-amyloid plaque accumulation in the cerebellum. A study by Murata et al., 2015 suggested that herbs like *Curcuma longa* and *Piper nigrum* as promising candidates for inhibiting the β-secretase enzyme. Besides, some phytomolecules like neferine (*Nelumbo nucifera*), ginsenoside Rg1 (*Panax notoginseng*), hispidin (*Phellinus linteus*), 2,2′,4′-trihydroxychalcone (*Glycyrrhiza glabra*), *etc.* showed promising outcomes as β-secretase inhibitors (Zhang and Tanzi, 2012). Because of its multifactorial nature, AD can adequately be managed through the “One-compound multiple-targets paradigm” treatment strategy. To overcome this limitation, herbal medicine could be a better approach, since they constitute the different classes of secondary metabolites targeting multiple signaling pathways or proteins, and show synergistic effects. The cholinesterase and β-secretase enzyme inhibitory potential of *M. oppositifolia* extract may serve as a good lead for the development of AD drug candidates.

Polypharmacy increases the risk of drug-related interactions leading to significant clinical complications. A study by Sonali et al., 2013 suggested that the co-administration of CYP3A4 and CYP2D6 interacting drugs not significantly might partially be involved in the plasma concentration of anti-AD drugs like donepezil to a clinically significant extent (Perry and Howes, 2011; Auxtero et al., 2021). So, it is important to collect consistent information on the safety of herbal products for their safe and effective use. The present finding indicated that the extract showed the least interaction potential against the CYP-isozymes compared to standard inhibitors and implies their safety aspects. Additionally, the quantified toxic heavy metals concentration in the plant extract was found to be well within the prescribed limits (Cd < 1 ppm, Pb < 1 ppm, Hg < 5 ppm) (Mukhopadhyay, 2018).

The UPLC-QTOF-MS analysis tentatively identified a total of 07 compounds from 07 different chemical classes. Genistein is an isoflavone distributed widely in the leguminous and fabaceae family. Isoflavone is well documented for its antioxidant, anti-inflammatory, and anti-cancer activities (Sharifi-Rad et al., 2021). *Mentha piperita* was reported to contain menthoside as one of the flavonoids reported for its antioxidant, and anti-inflammatory properties (Biswas et al., 2014). Ziguglycoside II from the root part of *Sanguisorba officinalis* was reported to have antioxidant properties (Byun et al., 2021).

The *in silico* molecular docking analysis showed menthoside as the most stable compound with a good docking score toward AChE, BChE and BACE-1 enzymes among all the identified metabolites. From binding site prediction the amino acids SER293 and TRP286 for AChE, GLY115 and TRP82 for BChE ARG128, SER36, PHE108, ASP228, VAL69, ARG128, and TYR71 for BACE-1 were found to be essential for the establishment of strong interaction.

The combination therapy concept has been explored as a novel approach in preventing and treating neurodegenerative conditions. The present findings suggested that *M. oppositifolia* could be effective in the development of lead against cholinesterase and β-Secretase enzymes useful in AD.

## Conclusion

Since global developments in the field of medication, no ultimate cure for Alzheimer’s disease has been recognized till date. Research from the past decades has come up with a few cholinergic drugs and NMDA receptor antagonists that do not affect the main pathological hallmarks of Alzheimer’s disease but rather somewhat delay the inevitable symptomatic disease progression. Being a multifactorial disease, there is a huge medical necessity for newer and more effective remedies that are free from deleterious side effects. It is noteworthy to study the ethnobotanical claim with high-throughput screening will cultivate novel therapeutic leads in drug development, thus contributing to a newer sight in the treatment of neurodegenerative disorders like Alzheimer’s disease. The findings concluded that *M. oppositifolia* could be a better optional pot in search of active moieties for multi-targeted drug approach due to its anti-cholinesterase and β-secretase enzyme inhibitory potential. The least cytotoxic effect on the HepG2 cell line and the cytochromeP450 isozyme inhibitory assay confirms its safety upon co-administration with CYP450 3A4 and 2D6 substrates drug candidates. The molecular docking analysis showed affinity and selectivity of the phytometabolites toward target enzymes. It can be said that herbs with rich polyphenols content might be preferable choices for the prevention of AD because of their multi-functions. Further detailed investigation is very much prerequisite to know the mechanistic approach toward the development of drugs against Alzheimer’s disease.

## Data Availability

The original contributions presented in the study are included in the article/Supplementary Material, further inquiries can be directed to the corresponding author.
