# Supplementary material for: Evaluation of Mollugo oppositifolia Linn. as cholinesterase and β-secretase enzymes inhibitor
Source: Front Pharmacol. 2023 Jan 4;13:990926. doi: 10.3389/fphar.2022.990926 (PMC9846241; doi:10.3389/fphar.2022.990926)
Supplement: Supplementary file 1 [file DataSheet1.docx]

**Figures:**

**
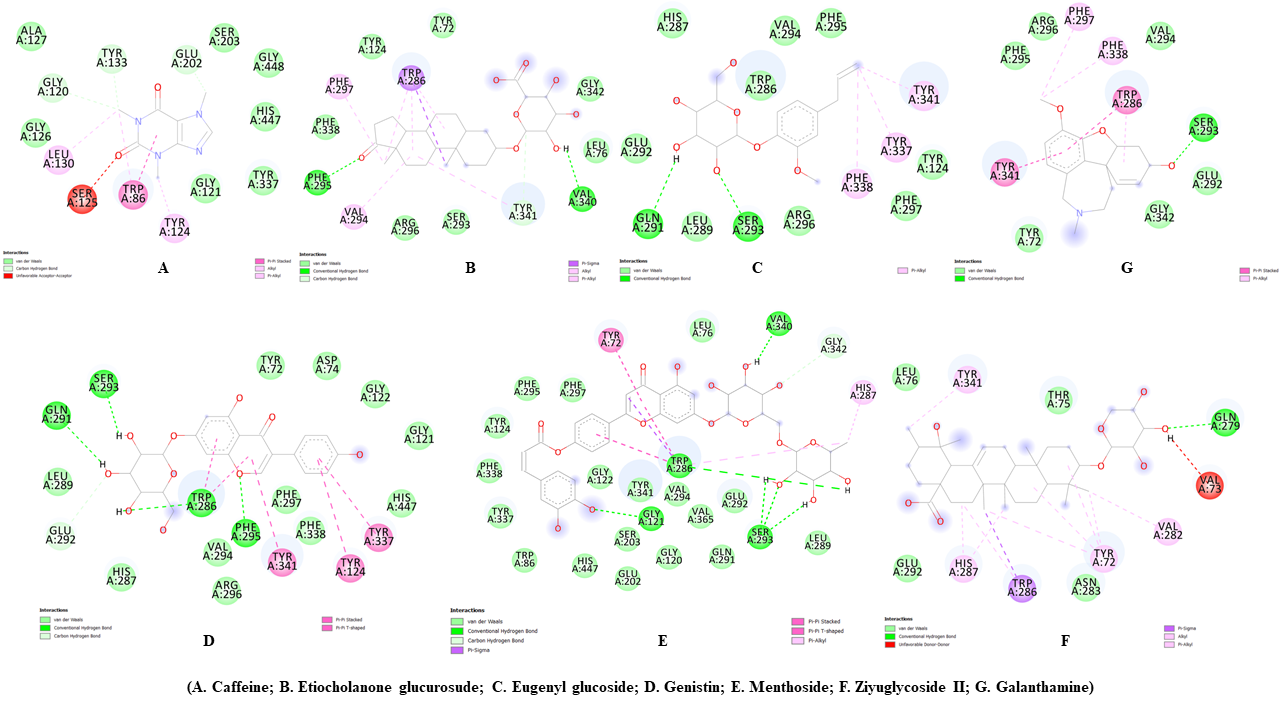
**

**Supplementary Figure S1:** 2D depiction showing various interactions of the phyto-compounds with AChE macromolecule (PDB ID: 4M0E).


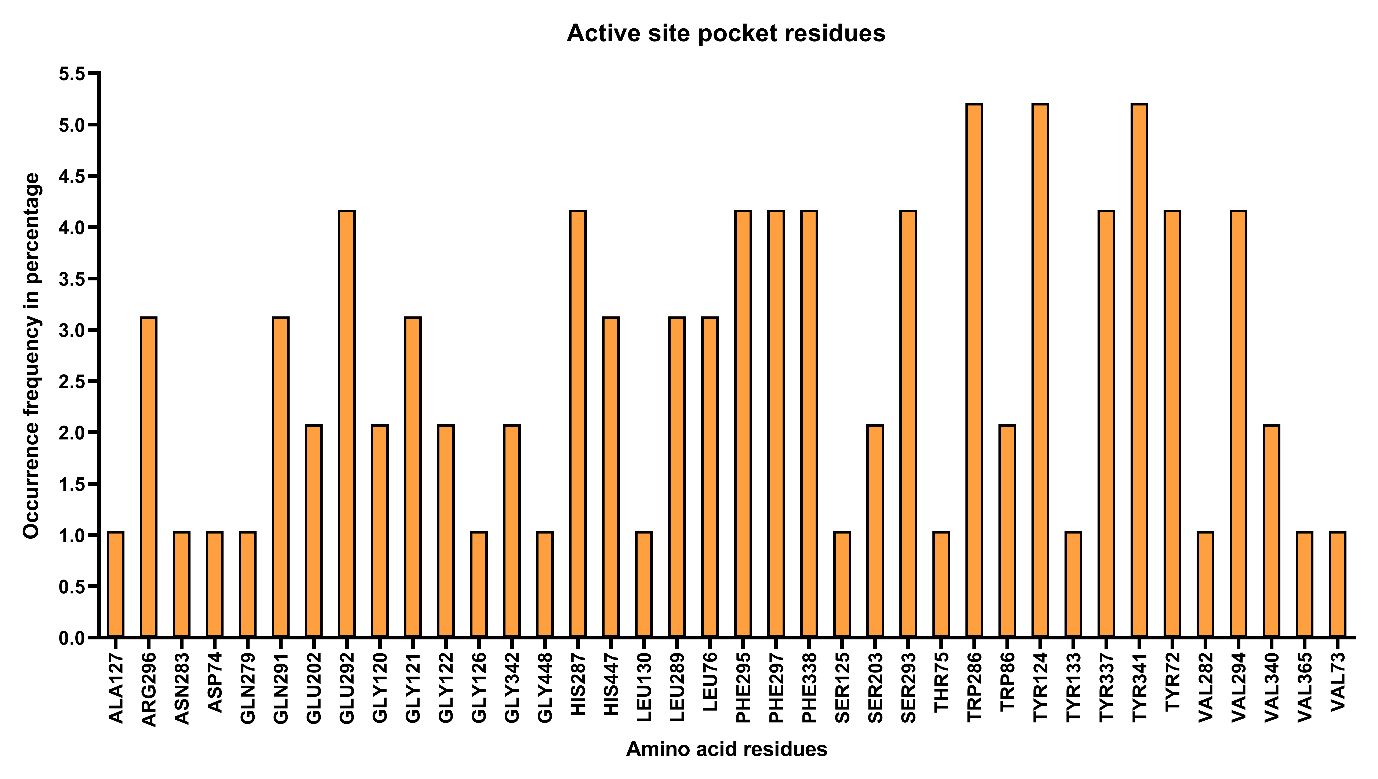


**Supplementary Figure S2:** The amino acid residues that interacted with the docked ligands and their frequency of occurrence in percentage.


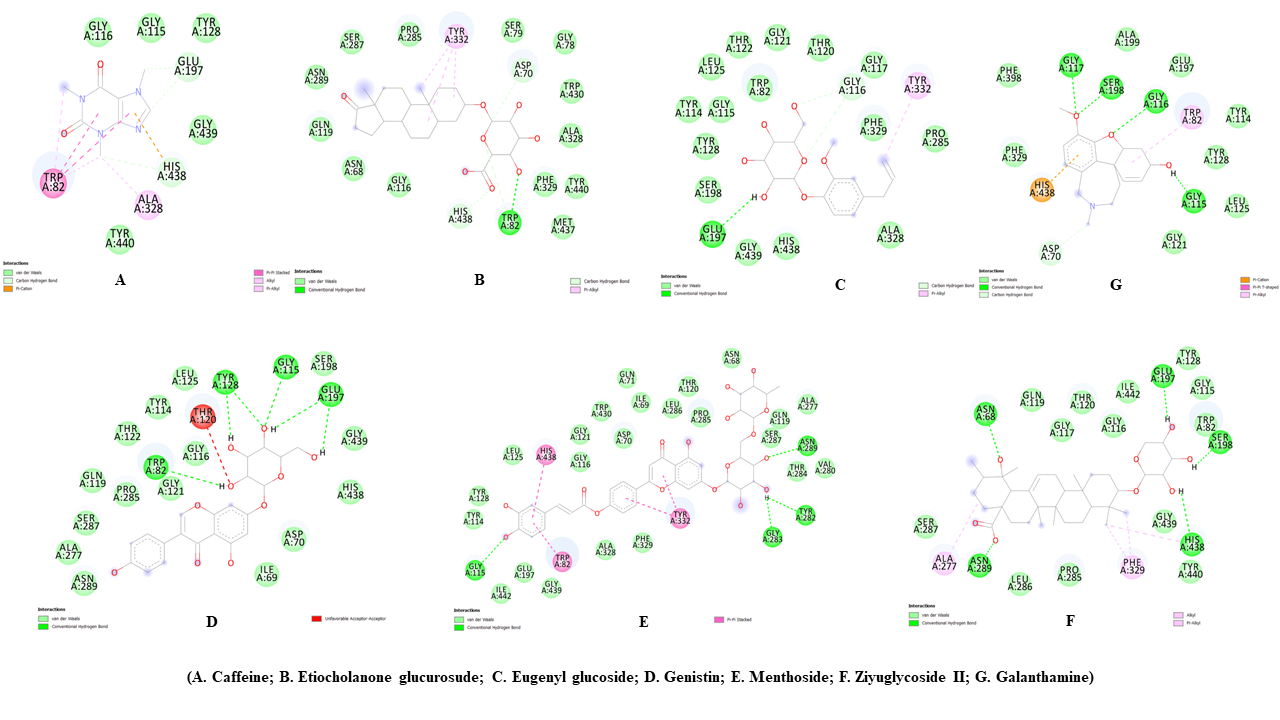


**Supplementary Figure S3:** 2D depiction showing various interactions of the phyto-compounds with BChE macromolecule (PDB ID: 5K5E).


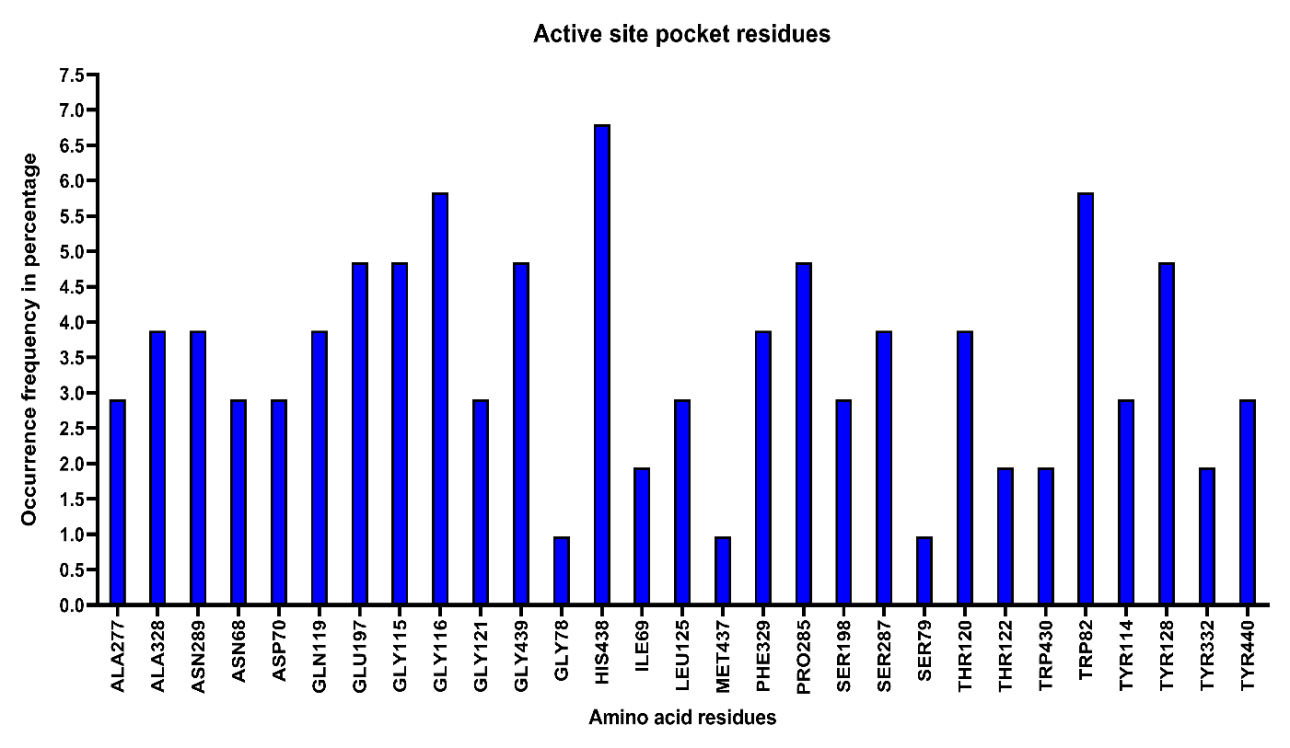


**Supplementary Figure S4:** The amino acid residues that interacted with the docked ligands and their frequency of occurrence in percentage.


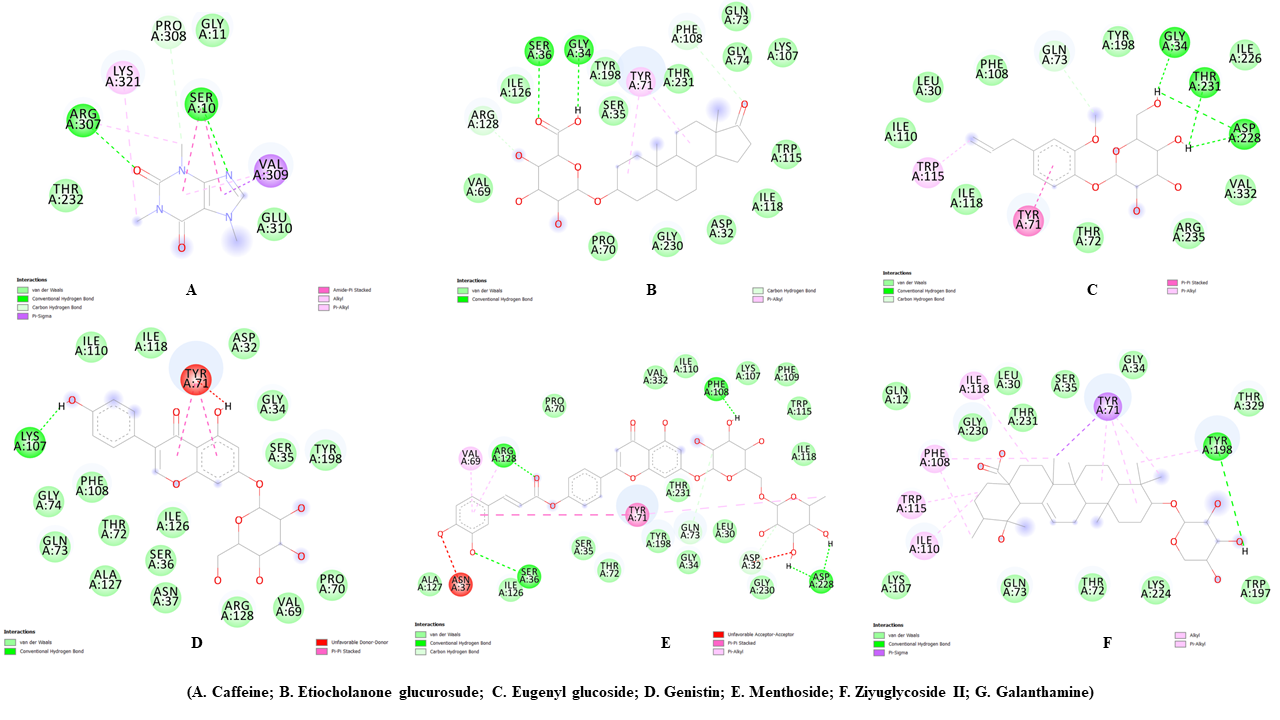


**Supplementary Figure S5:** 2D depiction showing various interactions of the phyto-compounds with BACE-1 macromolecule (PDB ID: 6OD6).


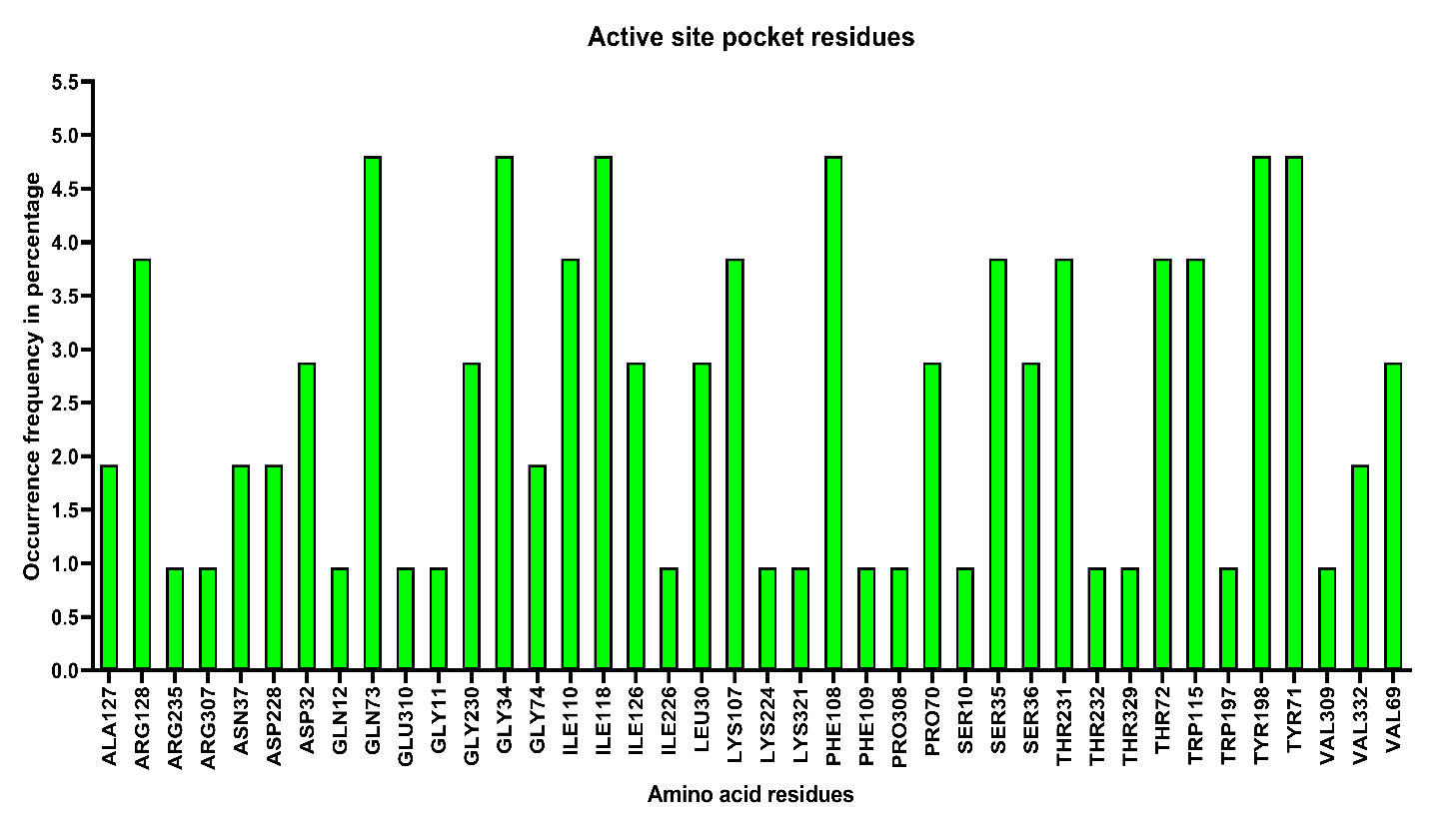


**Supplementary Figure S6:** The amino acid residues that interacted with the docked ligands and their frequency of occurrence in percentage.

**Tables:**

**Supplementary Table S1:** AAS instrumental condition for heavy metal analysis.

| **AAS specification** | **Element** | | |
| --- | --- | --- | --- |
|  | **Cadmium (Cd)** | **Lead (Pb)** | **Mercury (Hg)** |
| **Wavelength (nm)** | 228.8 | 217.0 | 253.7 |
| **Current** | 7.0 | 9.0 | 3.0 |
| **Flame** | AA | AA | AA |
| **Fuel (L min^-1^)** | 5.53 | 2.90 | 7.66 |
| **Slit width (nm)** | 0.5 | 0.5 | 0.5 |
| **Working range (ppm)** | 0.5-2.0 | 2.0-10.0 | 100-400 |
| **Read time (s)** | 3.0 | 3.0 | 3.0 |
| **Wash time (s)** | 10.0 | 10.0 | 10.0 |

**Supplementary Table S2:** Details of molecular docking studies of the phytoconstituents on AChE (PDB ID: 4M0E), which include Docking score, Conventional hydrogen bonds, Pi interactions, and active site pocket residues.

| **Phytocomponents** | **AChE activity** | | | |
| --- | --- | --- | --- | --- |
|  | **Docking Score** | **Conventional**  **-H Bond** | **Pi interaction** | **Active site pocket residues** |
| Etiocholanolone glucuronide | -8.5 | PHE295, VAL340 | TRP286(4), PHE297, VAL294, TYR341 | TYR72, TRP286, TYR124, PHE297, PHE338, PHE295, VAL294, ARG296, SER293, TYR341, VAL340, LEU76, GLY342 |
| Genistin | -9.4 | GLN291, SER293, TRP286, PHE295 | TYR341, TYR124, TYR337, TRP286(2) | SER293, GLN291, LEU289, GLU292, HIS287, TRP286, VAL294, ARG296, PHE295, TYR341, PHE338, PHE297, TYR124, TYR337, HIS447, GLY121, GLY122, ASP74, TYR72 |
| Menthoside | -11.1 | VAL340, TRP286, GLY121, SER293(3) | TYR72, HIS287, TRP286(4) | VAL340, TRP286, GLY121, SER293, TYR72, HIS287, PHE297, PHE295, TYR124, PHE338, TYR337, TRP86, HIS447, SER203, GLU202, GLY122, TYR341, GLY120, VAL294, VAL365, GLN291, GLU292, LEU289, GLY342, LEU76 |
| Caffeine | -6.5 | - | TRP86(2), TYR124, LEU130 | ALA127, GLY120, GLY126, LEU130, SER125, TRP86, TYR124, GLY121, TYR337, HIS447, GLY448, SER203, GLU202, TYR133 |
| Eugenyl glucoside | -7.9 | GLN291, SER293 | PHE338, TYR337, TYR341 | HIS287, GLU292, GLN291, LEU289, SER293, ARG296, PHE338, PHE297, TYR124, TYR337, TYR341, PHE295, VAL294, TRP286 |
| Ziyuglycoside II | -7.8 | GLN279 | TYR341, HIS287(2), TRP286(2), TYR72(4), VAL282 | GLN279, TYR341, HIS287, TRP286, TYR72, VAL282, THR75, LEU76, GLU292, ASN283, VAL73 |
| Galanthamine | -7.6 | SER293 | PHE297, PHE338, TRP286(2), TYR341 | SER293, PHE297, PHE338, TRP286, TYR341, PHE295, ARG296, VAL294, GLU292, GLY342, TYR72 |

**Supplementary Table S3:** Details of molecular docking studies of the phytoconstituents on BChE (PDB ID: 5K5E), which include Docking score, Conventional hydrogen bonds, Pi interactions, and active site pocket residues.

| **Phytocomponents** | **BChE activity** | | | |
| --- | --- | --- | --- | --- |
|  | **Docking Score** | **Conventional**  **-H Bond** | **Pi interaction** | **Active site pocket residues** |
| Etiocholanolone glucuronide | -9.2 | TRP82 | TYR332 (3) | TRP82, TYR332, PRO285, SER287, ASN289, GLN119, ASN68, GLY116, HIS438, MET437, TYR440, PHE329, ALA328, TRP430, ASP70, GLY78, SER79 |
| Genistin | -9.2 | TRP82, TYR128 (2), GLY115, GLU197 (2) | - | ASN289, ALA277, SER287, PRO285, GLN119, GLY121, GLY116, THR122, TYR114, LEU125, THR120, SER198, GLY439, HIS438, ASP70, ILE69, TRP82, TYR128, GLY115, GLU197 |
| Menthoside | -11.3 | GLY115, GLY283, TYR282, ASN289 | HIS438, TRP82, TYR332 (2) | ASN68, THR120, PRO285, LEU286, ILE69, GLN71, TRP430, ASP70, GLY121, GLY116, LEU125, TYR128, TYR114, ILE442, GLU197, GLY439, ALA328, PHE329, THR284, VAL280, SER287, GLN119, ALA277, GLY115, GLY283, TYR282, ASN289 HIS438, TRP82, TYR33 |
| Caffeine | -6.6 | - | TRP82 (4), ALA328 | TRP82, ALA328, GLY116, GLY115, TYR128, GLU197, GLY439, HIS438, TYR440 |
| Eugenyl glucoside | -7.9 | GLU197 | TYR332 | ALA328, PRO285, PHE329, GLY116, GLY117, THR120, GLY121, TRP82, THR122, LEU125, TYR114, GLY115, TYR128, SER198, GLY439, HIS438, GLU197, TYR332 |
| Ziyuglycoside II | -10.2 | ASN68, GLU197, SER198, HIS438, ASN289 | ALA277, PHE329 (2), HIS438 | GLN119, GLY117, THR120, GLY116, ILE442, TYR128, GLY115, TRP82, GLY439, TYR440, PRO285, LEU286, SER287, ASN68, GLU197, SER198, HIS438, ASN289, ALA277, PHE329, HIS438 |
| Galanthamine | -9.4 | GLY117, SER198, GLY115, GLY116 | TRP82 | GLY117, SER198, GLY115, GLY116, TRP82, ALA199, GLU197, TYR114, TYR128, LEU125, GLY121, ASP70, PHE329, PHE398 |

**Supplementary Table S4:** Details of molecular docking studies of the phytoconstituents on BACE-1 (PDB ID: 6OD6), which include Docking score, Conventional hydrogen bonds, Pi interactions, and active site pocket residues.

| **Phytocomponents** | **BACE-1 activity** | | | |
| --- | --- | --- | --- | --- |
|  | **Docking Score** | **Conventional**  **-H Bond** | **Pi interaction** | **Active site pocket residues** |
| Etiocholanolone glucuronide | -9.1 | SER36, GLY34 | TYR71 (2) | TYR198, SER35, THR231, PHE108, GLN73, GLY74, LYS107, TRP115, ILE118, ASP32, GLY230, PRO70, VAL69, ARG128, ILE126, SER36, GLY34, TYR71 |
| Genistin | -8.3 | LYS107 | TYR71 (2) | ILE110, ILE118, ASP32, GLY34, SER35, TYR198, PRO70, VAL69, ARG128, ILE126, SER36, ASN37, THR72, ALA127, PHE108, GLY74, GLN73, LYS107, TYR71 |
| Menthoside | -9.8 | ARG128, SER36, PHE108, ASP228 (2) | VAL69, ARG128, TYR71 (2) | ALA127, ASN37, ILE126, SER35, THR72, TYR198, THR231, GLN73, GLY34, LEU30, ASP32, GLY230, ILE118, TRP115, PHE109, LYS107, ILE110, VAL332, PRO70, ARG128, SER36, PHE108, ASP228, VAL69, ARG128, TYR71 |
| Caffeine | -5.1 | ARG307, SER10 | LYS321, ARG307, SER10 (2), VAL309 (2) | PRO308, GLY11, GLU310, THR232, LYS321, ARG307, SER10, VAL309 |
| Eugenyl glucoside | -6.3 | GLY34, THR231, ASP228 (2) | TRP115, TYR71 | ARG235, VAL332, ILE226, TYR198, GLN73, PHE108, LEU30, ILE110, ILE118, THR72, GLY34, THR231, ASP228, TRP115, TYR71 |
| Ziyuglycoside II | -9.4 | TYR198 | TYR198, TYR71 (4), ILE118, PHE108 (2), TRP115, ILE110 | GLN12, GLY230, LEU30, THR231, SER35, GLY34, THR329, TRP197, LYS224, THR72, GLN73, LYS107, TYR198, TYR71, ILE118, PHE108, TRP115, ILE110 |
